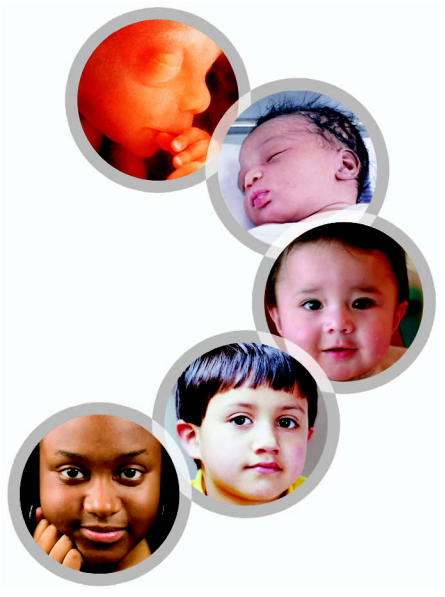# Children’s Health Centers: Past, Present, and Future

**DOI:** 10.1289/ehp.115-a192

**Published:** 2007-04

**Authors:** Angela Spivey

A physician who conducted some of the first studies documenting the effect of lead poisoning on children. An epidemiologist studying the effects of exposure to diethylstilbestrol (DES). A toxicologist pointing out the many different chemical exposures associated with impulsivity. Though each of their disciplines uses a different language, all of these scientists study the links between the environment and childhood disease. They are just a few of the researchers who converged at the “Children’s Environmental Health Research: Past, Present, and Future” conference held 22–23 January 2007 at the NIEHS. Sponsored in full by the NIEHS and planned jointly with the EPA, the conference aimed to get scientists talking about what types of research have been most effective. The result was recommendations to help chart the course for NIEHS children’s environmental health research over the next five to ten years, including the future of the Centers for Children’s Environmental Health and Disease Prevention Research.

## Maintaining a Critical Mass

Phil Landrigan, a pediatrician and chairman of community and preventive medicine at the Mount Sinai School of Medicine, who chaired the workshop, says the children’s research centers have created a critical mass of researchers in pediatrics, epidemiology, toxicology, human development, and other disciplines pursuing children’s health issues. “By virtue of having this critical mass, we were able in ten years to gain convergent toxicological and epidemiological i n f o r m a t i o n o n t h e developmental neurotoxicity of the organophosphate pesticides, [a process] that took nearly one hundred years to accomplish in the case of lead,” he says. In 2000, on the basis of this emerging science, the EPA banned residential use of the pesticides chlorpyrifos and diazinon, and research in the children’s centers has documented the success of community interventions to reduce children’s pesticide exposures using integrated pest management. “Another great advantage of the centers,” says Landrigan, “is that they are superb interdisciplinary incubators of the next generation of research leaders in children’s environmental health.”

Specific goals of the workshop included discussing recommendations for advancing research to home in on the contributions of the environment to disease in children, to develop better exposure and effects monitoring, to develop new strategies for intervention in and prevention of children’s diseases, and to translate research findings into clinical and public health practice.

The meeting was organized into sessions focusing on four case studies. The first discussions covered two cases in which epidemiological and toxicological research has led to successful interventions—lead neurotoxicity and asthma. Then scientists discussed whether lessons learned from these case studies may provide insight into the best approaches to take in addressing two emerging syndromes that may be included in future NIEHS research efforts—attention deficit/hyperactivity disorder (ADHD) and metabolic syndrome. Metabolic syndrome is defined by a cluster of three or more conditions, including high blood glucose, high blood pressure, elevated plasma triglyceride level, a low level of high-density lipoproteins (“good cholesterol”), and abdominal obesity.

Conference participants repeatedly pointed out the need to focus more on the basic mechanisms behind disease, rather than simply identifying epidemiological associations between exposures and disease. Identifying the mechanism behind an association helps speed up acceptance of a finding, says Chris Portier, NIEHS associate director for risk assessment. Several researchers also stressed the need to more systematically design studies so that epidemiological investigations and basic mechanistic studies coincide.

“We need to start looking at exposures that are able to be studied by both epidemiology and basic science,” says Theodore Slotkin, a professor of pharmacology and cancer biology at Duke University. “When those types of studies do overlap, it’s great, but we need to start connecting those things in a more organized way.”

Another topic that was raised repeatedly was the need for support of a major prospective epidemiological study of the effect of the environment on children’s health, such as the National Children’s Study. “Cohort studies are really invaluable because you’re measuring the exposure prior to any kind of outcome that occurs, so you avoid biases that a lot of other epidemiological study designs are prone to,” says Elizabeth Hatch, an associate professor of epidemiology at the Boston University School of Public Health.

Landrigan agrees that such a study is needed. “I see it as complementary to other more focused research programs, not replacing them,” he says. “Large prospective studies with banked biological specimens, archived historical information, and high-quality genetic data are extraordinarily powerful for identifying previously unrecognized etiologic associations and for elucidating synergies among environmental exposures as well as gene–environment interactions.”

In a similar vein, some workshop participants mentioned the need to increase the length of the funding cycle from the usual three to five years, to allow for more long-term, longitudinal studies. “That’s something that’s been under debate at NIEHS and NIH for some time,” Portier says.

## Complex Conditions

It has become apparent that just as asthma is a complex condition with many causes, so are metabolic syndrome and ADHD. In exploring possible causes, scientists are pinpointing not only current environmental factors such as diet but also early exposures, even before pregnancy, which may contribute to such conditions in offspring by disrupting hormones or early programming of growth and development.

As a culprit in the obesity epidemic, Robert Lustig, a professor of pediatric endocrinology at the University of California, San Francisco, pointed to high consumption of fructose, mainly in the form of man-made high-fructose corn syrup, found in a wide range of foods and drinks. Many scientists have pointed out that the rise in use of high-fructose corn syrup coincides with the obesity epidemic.

Lustig suggested some possible mechanisms. Unlike glucose, fructose doesn’t stimulate insulin production, and it does not produce feelings of fullness when consumed, because it doesn’t suppress ghrelin, a hormone that is thought to stimulate hunger (many of these findings are reviewed by Yuren Wei of Colorado State University and colleagues in the January 2007 *Journal of Nutritional Biochemistry*). Lustig also pointed to a study in the July 2005 issue of *Diabetes* in which David Faeh of the University of Lausanne, Switzerland, showed that normal male adults who were overfed fructose for six days developed several features of metabolic syndrome. These included increases in triglycerides and *de novo* lipogenesis—the conversion of carbohydrates into fat.

Amanda Drake, a clinician/scientist at the University of Edinburgh who studies early-life origins of disease, pointed to prenatal targets that, if perturbed at the right time, may predispose offspring to obesity and associated conditions through programming of the growth and development of tissues or of the brain. For instance, she discussed studies that have shown a strong link between low birth weight and impaired glucose tolerance, type II diabetes, dyslipidemia, and metabolic syndrome. Such studies suggest that these conditions may be modulated by postnatal growth, Drake says. That is, during accelerated postnatal growth—a compensation for low birth weight—the body may switch on processes that incline the body toward obesity and associated conditions.

The wide-ranging perspectives of the participants became apparent as they discussed which avenues of study may most quickly determine mechanisms behind associations. Some suggested there is value in studying a single toxicant and trying to elucidate all its effects, as was done with lead. “What would have happened with lead if we hadn’t focused on all the symptoms of exposure? We may be limiting what we know by focusing only on, for example, ADHD [to the exclusion of other conditions],” says Slotkin. He suggested that while funding work on a specific disease such as ADHD is a good way to stimulate research, programs should be flexible enough that their direction can be broadened based on new findings.

In the session on metabolic syndrome, with regards to obesity in particular, one candidate that emerged for study was the synthetic estrogen DES because it has been used as a model for potential effects of endocrine disruptors. For example, in the July 2005 *Birth Defects Research Part A: Clinical and Molecular Teratology*, Retha Newbold and colleagues from the NIEHS found that mice exposed to low doses of DES during pregnancy produced offspring that became overweight later in life. Although there are no human studies to date of the effect of DES on weight, findings on other effects of the compound in humans have paralleled what has been found in animals, Hatch said. She is exploring possible associations between DES exposure and obesity in humans by collecting data on body mass index and waist circumference in an ongoing National Cancer Institute–funded study of DES-exposed and unexposed women.

Other researchers pointed to the need to better understand the common pathways that may be affected by many different exposures. In the session on ADHD, Jason Richardson, an assistant professor of environmental and occupational medicine at the University of Medicine and Dentistry of New Jersey–Robert Wood Johnson Medical School, pointed to recent studies suggesting that different toxicants, perhaps acting at different key time points, are associated with one particular behavior—impulsivity. For instance, in the December 2006 issue of *EHP*, Paul Stewart and colleagues showed that children with either prenatal polychlorinated biphenyl (PCB) exposure, postnatal lead exposure, or prenatal methylmercury exposure all showed increased impulsivity.

“We’re starting to see that it may be that divergent compounds converge upon common pathways and may lead to impulsivity not only in animal models but in the human population,” Richardson says. “Instead of looking at one toxicant at a time, maybe we can use these common pathways to really try to pin down mechanisms by which divergent compounds produce common behavioral effects.”

Such exposures may act on several systems in the brain. There is firm evidence for their action on the dopamine system; for example, in the August 2006 issue of *Toxicological Sciences*, Richardson and colleagues including Mike Caudle of Emory University showed that PCB exposure disrupts dopamine transport in mice. In addition, in the November 2002 issue of *EHP*, Richard Seegal of the New York State Department of Health showed that exposure of adult rats to low concentrations of PCBs initially increased concentrations of circulating dopamine, but significantly decreased them after three days.

“Based on the documented effects of certain developmental neurotoxicants on the dopamine system and the link between dopaminergic alterations and behavioral deficits, such as impulsive-like behaviors, this appears to be a promising avenue for investigation in order to understand both the effects of chemical exposure on the nervous system and how these effects translate into behavioral abnormalities,” Richardson says.

## What’s Next for the Centers

Among the conference attendees was a committee of senior scientists charged with reviewing the Centers for Children’s Environmental Health and Disease Prevention Research program and related investigator-initiated research on children’s health. After meeting in separate evening sessions during the workshop, the committee drafted a report that was released for public comment in mid-March 2007.

The review committee has expressed broad and strong support for the children’s center mechanism to continue, according to Kim Boekelheide, a professor of pathology and lab medicine at Brown University and a member of the committee. “It’s an important mechanism that provides an interdisciplinary approach to what is a very important problem,” he says. The committee also generally agreed on the need to strengthen the centers’ basic-mechanisms research component, while keeping programs focused on actual children’s health issues.

After a 30-day public comment period, the science advisory board of the NIEHS—the National Advisory Environmental Health Sciences Council—will discuss the review committee’s report and comments in a public teleconference. In May, after considering the board’s recommendations, NIEHS director David Schwartz will propose how the institute should move forward in funding children’s environmental health research, says Portier.

The NIEHS would fund research along many different avenues as long as the proposal was highly rated scientifically, according to Portier. “We wouldn’t rule anything out,” he says, “because obviously we want the best science we can possibly get.”

## Figures and Tables

**Figure:**